# Shu-Xie decoction alleviates oxidative stress and colon injury in acute sleep-deprived mice by suppressing p62/KEAP1/NRF2/HO1/NQO1 signaling

**DOI:** 10.3389/fphar.2023.1107507

**Published:** 2023-02-06

**Authors:** Mengyuan Wang, Bo Li, Yijiang Liu, Mengting Zhang, Caoxin Huang, Teng Cai, Yibing Jia, Xiaoqing Huang, Hongfei Ke, Suhuan Liu, Shuyu Yang

**Affiliations:** ^1^ Research Studio of Traditional Chinese Medicine, The First Affiliated Hospital of Xiamen University, School of Medicine, Xiamen University, Xiamen, Fujian, China; ^2^ The First Affiliated Hospital of Xiamen University, School of Medicine, Xiamen University, Xiamen, Fujian, China; ^3^ Xiamen Diabetes Institute, The First Affiliated Hospital of Xiamen University, School of Medicine, Xiamen University, Xiamen, Fujian, China; ^4^ Research Center for Translational Medicine, The First Affiliated Hospital of Xiamen University, School of Medicine, Xiamen University, Xiamen, Fujian, China

**Keywords:** sleep deprivation, oxidative stress, NRF2, traditional Chinese medicine, ROS

## Abstract

**Introduction:** Sleep disorders are common clinical psychosomatic disorders that can co-exist with a variety of conditions. In humans and animal models, sleep deprivation (SD) is closely related with gastrointestinal diseases. Shu-Xie Decoction (SX) is a traditional Chinese medicine (TCM) with anti-nociceptive, anti-inflammatory, and antidepressant properties. SX is effective in the clinic for treating patients with abnormal sleep and/or gastrointestinal disorders, but the underlying mechanisms are not known. This study investigated the mechanisms by which SX alleviates SD-induced colon injury *in vivo*.

**Methods:** C57BL/6 mice were placed on an automated sleep deprivation system for 72 h to generate an acute sleep deprivation (ASD) model, and low-dose SX (SXL), high-dose SX (SXH), or S-zopiclone (S-z) as a positive control using the oral gavage were given during the whole ASD-induced period for one time each day. The colon length was measured and the colon morphology was visualized using hematoxylin and eosin (H&E) staining. ROS and the redox biomarkers include reduced glutathione (GSH), malondialdehyde (MDA), and superoxide dismutase (SOD) were detected. Quantitative real-time PCR (qRT-PCR), molecular docking, immunofluorescence and western blotting assays were performed to detect the antioxidant signaling pathways*.*

**Results:** ASD significantly increased FBG levels, decreased colon length, moderately increased the infiltration of inflammatory cells in the colon mucosa, altered the colon mucosal structure, increased the levels of ROS, GSH, MDA, and SOD activity compared with the controls. These adverse effects were significantly alleviated by SX treatment. ASD induced nuclear translocation of NRF2 in the colon mucosal cells and increased the expression levels of p62, NQO1, and HO1 transcripts and proteins, but these effects were reversed by SX treatment.

**Conclusion:** SX decoction ameliorated ASD-induced oxidative stress and colon injury by suppressing the p62/KEAP1/NRF2/HO1/NQO1 signaling pathway. In conclusion, combined clinical experience, SX may be a promising drug for sleep disorder combined with colitis.

## 1 Introduction

Sleep homeostasis is critical for human health and metabolism (Xie et al., 2013). Sleep-wake cycles regulate several biological functions associated with the gastrointestinal tract including waste clearance, cell repair and regeneration, digestion, absorption, and electrolyte balance (Hablitz et al., 2020). Sleep deprivation (SD) is defined as less than 6 h sleep per night, and is caused by lifestyle changes, aging, and disordered sleep (Siegel, 2022). Individuals with SD exhibit physical stress and aberrant physiological functions (Musiek et al., 2018). SD disrupts neuro-autonomic control (Deurveilher et al., 2021), inflammatory and coagulation pathway responses, and increases oxidative stress. Therefore, SD causes metabolism disorders (Pandey and Kar, 2018), digestive diseases, cardiovascular diseases (Shamsuzzaman et al., 2003), and neurological disorders (Cappuccio et al., 2010; Patel et al., 2012; Orr et al., 2020).

Oxidative stress is caused by an imbalance between the production of reactive oxygen species (ROS) and the activity of antioxidant mechanisms (Sies et al., 2017). ROS including superoxide, hydrogen peroxide, hydroxyl radical, and nitric oxide are highly reactive, and are formed in cells due to incomplete reduction of molecular oxygen during metabolic activities. The peroxyl radicals are highly reactive and disrupt the integrity of the membranes by oxidizing integral proteins and polyunsaturated lipids in the cell membranes (Folden et al., 2003). Malondialdehyde (MDA) is the end product of lipid peroxidation and is an effective biomarker of lipid peroxidation and an indirect index of the reactive oxygen species (ROS) levels in the plasma (Weismann et al., 2011). The enzymatic and non-enzymatic antioxidant mechanisms, including α-tocopherol (Wallert et al., 2019), ascorbate (Goncalves et al., 2021), uric acid (Amaro et al., 2015), catalase (Ren et al., 2018), superoxide dismutase (SOD) (Zhang et al., 2018a), and reduced glutathione (GSH) (Wang et al., 2021a) protect the cells against the deleterious effects of free radicals and lipid peroxidation. The cellular stress response to acute sleep deprivation involves ROS generation due to oxidative stress and activation of antioxidant mechanisms and enzymes to detoxify ROS (Ramanathan and Siegel, 2011; Vaccaro et al., 2020; Koutsoumparis et al., 2022). Sleep deprivation (SD) induces ROS accumulation (Pandey and Kar, 2018) in various tissues including the colon (Vaccaro et al., 2020). Furthermore, SD-induced pathology in the colon is alleviated by scavenging the free radicals by inducing the antioxidant mechanisms (Alzoubi et al., 2019).

*SQSTM1* gene encodes p62, a multifunctional ubiquitination-binding scaffold protein, which shuttles between the nucleus and the cytoplasm (Kwon et al., 2019). p62 participates in the ubiquitin-proteasomal and the autophagy-lysosomal protein degradation systems, and plays a key role in DNA repair and oxidative stress responses (Kwon et al., 2019). Aberrant expression of p62 is reported in carcinogenesis (Wei et al., 2014; Moscat et al., 2016), neurodegenerative disorders (Calvo-Garrido et al., 2019; Blaudin de Thé et al., 2021), and metabolic diseases such as obesity (Aragonès et al., 2020), T2DM, and NAFLD (Hu and Zender, 2016).

Nuclear factor erythroid 2-related factor 2 (NRF2) is a key player in the cellular antioxidant response mechanisms (Pan et al., 2016; Singh et al., 2019). During normal conditions, Kelch-like ECH-associated protein 1 (KEAP1) associates with NRF2 (Hochmuth et al., 2011; Hast et al., 2013) and promotes its degradation through the ubiquitin proteasome pathway (Villeneuve et al., 2013). Oxidative stress induces oxidation of KEAP1 at multiple cysteine residues (Dayalan Naidu et al., 2018; Suzuki et al., 2019). This disrupts association between KEAP1 and NRF2. Furthermore, during oxidative stress, p62 binds to KEAP1, induces ubiquitination, and subsequent degradation of KEAP1 (Fan et al., 2010; Komatsu et al., 2010). Therefore, under sustained oxidative stress conditions with elevated ROS, newly synthesized NRF2 protein molecules translocate to the nucleus, bind to the AREs of antioxidant response genes, and drive the transcription of antioxidant genes such as NAD(P)H quinone oxidoreductase 1 (NQO1) and heme oxygenase 1 (HO1) (Huang et al., 2000; Eggler et al., 2009).

Clinical treatment for sleep disorders includes drugs such as hypnotic benzodiazepines (temazepam and nitrazepam), Z drugs (zolpidem and zopiclone), and melatonin. These drugs provide temporary relief from SD-related symptoms but are also associated with multiple adverse effects. The (S)-enantiomer of zopiclone (eszopiclone, S-z) is a non-benzodiazepine cyclopyrrolone, which significantly improves sleep efficiency, sleep latency, wake time after sleep onset, number of awakenings, number of nights awakened weekly, total sleep time, quality of sleep, and depth of sleep compared with the placebo (*p* < 0.05) (McCall et al., 2006). Eszopiclone is commonly prescribed for insomnia (Najib, 2006). An overdose of zopiclone, either alone or together with its metabolites, causes oxidative stress in the erythrocytes, and is associated with adverse effects such as methemoglobinemia and hemolytic anemia (Chan, 2014).

Herbal medicine is commonly used for the treatment of a variety of gastrointestinal disorders (Kong et al., 2020; Molagoda et al., 2020; Wu et al., 2021). Shu-Xie Decoction (SX) is an herbal formula developed by Dr. Shuyu Yang and colleagues using a combination of Suanzaoren Decoction and Huanglian Jiedu Decoction, also called Yangxue Rougan decoction. The quality and composition of the SX decoction was confirmed by UHPLC-QE-MS (Zhang et al., 2022). SX is used in the First Affiliated Hospital of Xiamen University (Xiamen, China) for the management of diabetic patients with sleep disturbances, mood disorders, and gastrointestinal dysfunction, which are collectively referred to as a triad of ‘Shu-Xie.’ A previous study reported that SX suppresses inflammation and depression in mice (Zhang et al., 2022). However, the underlying mechanisms by which SX protects against SD-induced colon dysfunction are poorly understood.

Network pharmacology combines high-throughput data integration (Kamdar and Musen, 2017), database searching and mining (Barneh et al., 2016), target prediction (Tanoli et al., 2018), and simulation laboratories (Hsin et al., 2016). Its systematic and holistic nature is in line with the principles of holistic view and evidence-based treatment of TCM, which can effectively promote the in-depth study of TCM compounding and reveal the material basis of the efficacy of TCM (Xu et al., 2019). The research idea and method are to firstly screen the main active ingredients and targets of the herbal compound, then collect and screen the therapeutic targets of the herbal compound, construct the target interaction network between the herbal compound and the disease, conduct protein interaction analysis, and gene function enrichment analysis. Finally, molecular docking will be performed to reveal the targets and molecular mechanisms of the herbal compound for the treatment of certain diseases.

Therefore, in this study, we investigated the clinical value of SX in alleviating SD-induced colon dysfunction and the underlying mechanisms using a mouse model. Our aim was to provide pre-clinical evidence for the utility of SX in the clinical treatment of patients with sleep deprivation-related disorders (Wang et al., 2021b).

## 2 Materials and methods

### 2.1 Animals

Six-to-eight-week-old SPF-grade male C57BL/6J mice (20 ± 1 g) were purchased from Beijing Vital River Laboratory Animal Technology Co., Ltd. (Beijing, China), and housed at the Laboratory Animal Center of Xiamen University (Xiamen University, Fujian, China) according to the institutional guidelines. The mice were maintained under ambient laboratory conditions, which included 55 ± 5% relative humidity (RH), 22°C ± 2°C temperature, and a 12:12 h light: dark cycle. They were fed a standard chow diet (SDS RN3) and filtered water. All the animal experiments were approved by the Animal Ethics Committee of Xiamen University (Acceptance No.: XMULAC20200142).

### 2.2 Preparation of the SX decoction

Shu-Xie Decoction (SX) was prepared from 10 plant materials (Supplementary Table S1) by the TCM Pharmacy of the Nanputuo Branch, The First Affiliated Hospital of Xiamen University (Xiamen, China). The low and high doses of the SX Decoction (1.56 g/mL and 3.12 g/mL) were prepared for animal administration using a rotary evaporation apparatus at 45°C. The dosage was based on the oral SX dose for humans (1.714 g/kg per day). Therefore, the dosage was 15.6 g/kg per day (low dose) and 31.2 g/kg per day (high dose) for mice. The intragastric dosing for mice was set at 0.1 mL/10 g body weight. SX dosing in the 2 doses were administered by oral gavage once a day for 3 days during the modeling period.

### 2.3 S-zopiclone

S-zopiclone (S-z) was donated by the First Affiliated Hospital of Xiamen University (Xiamen, China). The intragastric dosing of mice was 3.75 mg/kg/day or 0.375 mg/mL (2 mg S-z dissolved in 5.33 mL distilled water) as previously published (Gerashchenko D and Kilduff, 2017).

### 2.4 Animal grouping and model construction

**FIGURE 1:**
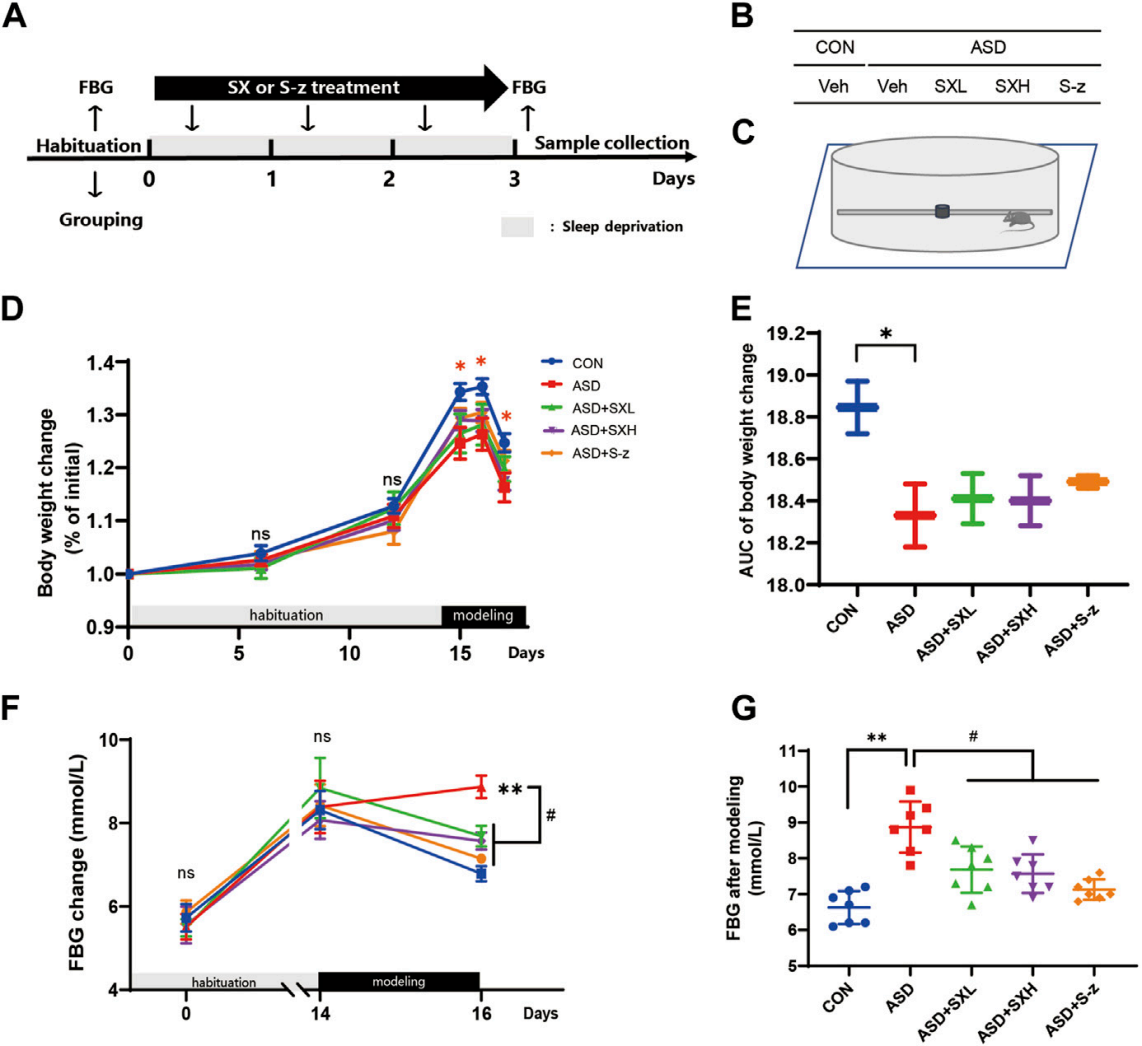
SX alleviates ASD-induced clinical symptoms associated with gastrointestinal dysfunction in mice. **(A)** Schematic representation of the experimental design. After 14 days of habituation, mice were sleep deprived for 3 days and orally administered with distilled water, SX, or S-z, once a day. Fasting blood glucose levels, body weight, and food and water consumption were recorded at all points. **(B)** Grouping of mice. CON = control; ASD = acute sleep deprived; Veh = vehicle; SXL = low-dose SX; SXH = high-dose SX; S-z = S-zopiclone. **(C)** Schematic diagram shows acute sleep deprivation modeling. SX alleviates ASD-induced weight loss. **(D, E)** Body weights of the CON, ASD, ASD + SXL, ASD + SXH, and ASD + S-z group mice during habituation and ASD modeling. *p <* 0.05, CON vs. ASD; **(F, G)** Fasting blood glucose levels of the CON, ASD, ASD + SXL, ASD + SXH, and ASD + S-z group mice during habituation and ASD modeling. *p <* 0.05, ASD vs. SXL, SXH and S-z. Data were expressed as means ± SEM. *n* = 7 per group. Two-way ANOVA for D and F; one-way ANOVA for E and G; ^*^ *p* < 0.05, ^**^ *p* < 0.01 vs. control group; ^#^ *p* < 0.05 vs. ASD group; ns, no significant.

The C57BL/6J mice (*n* = 50) were randomly assigned to the following five groups (n = 10 per group) and allowed to acclimatize for 1–2 weeks: 1) normal control (CON) group (the non-sleep deprived group; provided distilled water; mice placed in the restriction chamber without rotating bar); 2) ASD group (24 h sleep deprivation for 3 days; provided with distilled water; placed in the restriction chamber); 3) ASD + SXL group (24 h sleep deprivation for 3 days; 11.5 g/kg/d SX decoction; placed in the restriction chamber); 4) ASD + SXH group (24 h sleep deprivation for 3 days; 23 g/kg/d SX decoction; placed in the restriction chamber); and 5) ASD + S-z group (24 h of sleep deprivation for 3 days; 3.75 mg/kg S-zopiclone; placed in the restriction chamber) (Figure 1B). SD was performed using the horizontal bar-style automated sleep deprivation system for mice (YAN-239, Shanghai Xiyao Biological Technology Co., LTD, Shanghai, China; bar speed: 5; rotation direction changed every 10–30 s; stopped for 1 min after every 5 min) (Figure 1C). While modeling, the control and the ASD group mice were provided with the same amount of distilled water daily *via* gavage. All the treatment groups were provided with the appropriate therapeutic drug using the gavage between 8:00 a.m. and 10:00 a.m., once a day for three consecutive days. The body weights and food and water intake were estimated daily. The study protocol is shown in Figure 1A.

### 2.5 Sample collection

The mice were subjected to fasting during the last 10 h of modeling. Then, the mice were anesthetized with isoflurane and weighed. The blood samples were collected using retro-orbital bleeding method. After sacrificing the mice, the colons were carefully harvested and weighed. The colon length was measured. The colons were separated from the mesenteric and adipose tissues, cut along the longitudinal axis, and rinsed with ice-cold phosphate-buffered solution (pH = 7.4). The colons of four mice from each group were Swiss rolled and fixed in neutral buffer containing 10% formalin for 24 h at 2°C–8°C. The fixed colon tissues were dehydrated with gradient ethanol solutions, and embedded in paraffin at 56°C–60°C for further histological and immunofluorescence analysis. The colons of the remaining six mice in each group were frozen quickly in liquid nitrogen and stored at −80°C. The blood samples were centrifuged at 12,000 rpm and room temperature for 5 min. The serum and erythrocyte samples were collected for further analysis.

### 2.6 Histopathology

The paraffin-embedded colon Swiss rolls were cut into 4-μm-thick slices (RM2245, Leica, German) and stained with hematoxylin and eosin (H&E) (C0105S, Beyotime Biotechnology, Shanghai, China). The stained sections were photographed using the Leica Aperio micro-scanning system (Versa 200, Leica, German) and scored to quantify the degree of intestinal injury using a previously published scoring system by Ding and Wen, which included scoring the degree of intestinal epithelial cell damage and the degree of inflammatory infiltration (Ding and Wen, 2018).

### 2.7 Assay for ROS

ROS levels in blood cells were measured for three consecutive days using the DCFDA/H2DCFDA- Cellular ROS Assay Kit (ab113851, Abcam, United States) according to the manufacturer’s instructions. DCFH-DA was diluted 1:1,000 with PBS to obtain a final concentration of 10 μmol/L. Blood cells (200,000 cells per 0.1 mL) were incubated for 30 min with diluted DCFH-DA in a 37°C cell incubator with constant mixing every 3–5 min. The cells were washed three times with serum-free medium to remove excess DCFH-DA. The fluorescence intensity in the stained cells was measured at 485/535 nm using a digital microplate luminometer (Varioskan Flash Microplate Reader, Thermo Fisher Scientific, United States).

### 2.8 Estimation of serum GSH, SOD activity, and MDA levels

Serum GSH levels were estimated using the GSH Assay Kit (A006-2; Nanjingjiancheng, Nanjing, China) according to the manufacturer’s instructions. Total SOD activity in the serum was estimated using the SOD activity assay kit (A001-3; Nanjingjiancheng, Nanjing, China) according to the manufacturer’s instructions. The levels of serum MDA, a by-product of lipid peroxidation, were estimated using the Lipid Peroxidation MDA Assay Kit (Beyotime Biotechnology, Shanghai, China) according to the manufacturer’s instructions.

### 2.9 AutoDock and Autodock Vina analysis

The TCMSP database was used to obtain the main active ingredients and targets of SX (Ru et al., 2014), then the therapeutic targets of SX were collected and screened, and the “Cytoscape” software was used to build a network of SX and ASD target interactions to find the drug components that interacted most with the targets (Su et al., 2014). Protein interactions were analyzed through the STRING website, as well as functional GO and KEGG enrichment analyses (Szklarczyk et al., 2011; Kanehisa et al., 2017). The 2D planar structures of Baicalin (BAI) and Paeoniflorin (PAE) were downloaded from the ChemSpider website (www.chemspider.com) and converted to 3D spatial structures using the OpenBabel (version 3.1.1) toolbox (O'Boyle et al., 2011). The sequences of p62, KEAP1, NRF2, NQO1, and HO1 proteins were downloaded from Protein Data Bank (https://www.rcsb.org/), processed by the Pymol software (Seeliger and de Groot, 2010), and docked onto the 3D structures of BAI and PAE proteins using the AutoDockTools (1.5.7) to determine their binding efficiencies. The compounds with the lowest binding energy were screened by molecular docking to reveal the targets. The binding affinity or binding energy values less than 0 indicated spontaneous binding of the ligand to the receptor.

### 2.10 Western blotting

The colon tissues were incubated with the RIPA lysis buffer (Merck Millipore) containing 1 mM Phosphatase inhibitor and protease inhibitor cocktail (ThermoFisher), ground in a tissue grinder, lysed on ice for 30 min, and centrifuged at 12,000 rpm and 4°C for 10 min. The supernatant was collected and the protein concentrations of the samples were quantified using the BCA protein assay kit (ThermoFisher, United States). Equal amounts of protein samples (30 mg/sample) were denatured by boiling in the loading buffer containing bromophenol blue for 10 min. Then, the samples were separated on a 10% SDS-polyacrylamide gel electrophoresis (SDS-PAGE) and transferred onto a 0.45 μm polyvinylidene fluoride (PVDF) membrane (Millipore, MA, United States). The membranes were blocked with 5% BSA in 1 × PBST for 1.5 h at room temperature and incubated overnight at 4°C with primary antibodies against p62, KEAP1, NRF2, NQO1, HO1, and β-actin (Supplementary Table S2) on a shaker. Then, after washing in 1 × PBST, the blots were incubated with HRP-conjugated secondary antibody (ThermoFisher, United States) at room temperature for 1 h. The blots were then developed using the Western Bright ECL chemiluminescent substrate kit (Advansta Inc., San Jose, CA, United States). The protein bands were visualized and analyzed using the Qinxiang imaging system (Clinx Science Instruments Co. Ltd., Shanghai, China).

### 2.11 Real-time reverse transcription-polymerase chain reaction

Total RNA was extracted from the colon samples (50 mg per mice) using the RNA Extraction kit (DP419, Tiangen, Beijing, China) according to the manufacturer’s protocol. The RNA samples were quantified using the Nanodrop spectrophotometer (ND-1000, Nanodrop Technologies, United States). Then, cDNA templates were prepared from RNA samples (2000 ng per sample) using the FastKing RT kit (with gDNase) (KR116, Tiangen, Beijing, China). Q-PCR analysis was performed in a Real-time PCR machine (LightCycler®480 II, Roche, Swiss) using specific primer sequences (Supplementary Table S3) and SuperReal PreMix Plus kit (SYBR Green) (FP205, Tiangen, Beijing, China). Relative mRNA levels of specific genes were estimated using the 2^−ΔΔCT^ method.

### 2.12 Immunofluorescence

Paraffin-embedded colonic Swiss rolls were cut into 4-μm-thick sections, washed three times with 0.01 M PBS (pH = 8.0), and blocked and permeabilized in 1 × PBS containing 5% BSA and 0.02% TritonX-100 for 1 h at room temperature. Then, the sections were incubated with primary antibodies against HO1 (1:100), NQO1 (1:100), and NRF2 (1:100) in a wet and dark box device overnight at 4°C. Later, the sections were washed with PBS, placed in a dark box and incubated with Alexa Fluor 488 or 594-conjugated secondary antibodies (Invitrogen, 1:200, United States) at room temperature for 1 h. Mouse IgG was used as the negative control. The sections were then washed with PBS and sealed with anti-fluorescence quenching sealing medium containing DAPI (Vector Laboratories, Newark, California, United States). The stained samples were imaged at × 200 and × 400 magnification using the Nikon Eclipse C1 Ortho-Fluorescent Microscope (Nikon, Japan).

### 2.13 Statistical analysis

The statistical analysis was performed using the GraphPad Prism software, version 8.0 (GraphPad, CA, United States). The data are represented as means ± SEM. The data from multiple groups were compared using the one-way analysis of variance (ANOVA) comparison test. Two-way Repeated Measures ANOVA was used to compare the data between repeated measures at various times. *p* < 0.05 was considered as statistically significant.

## 3 Results

### 3.1 SX alleviates ASD-induced clinical symptoms in mice

**FIGURE 2:**
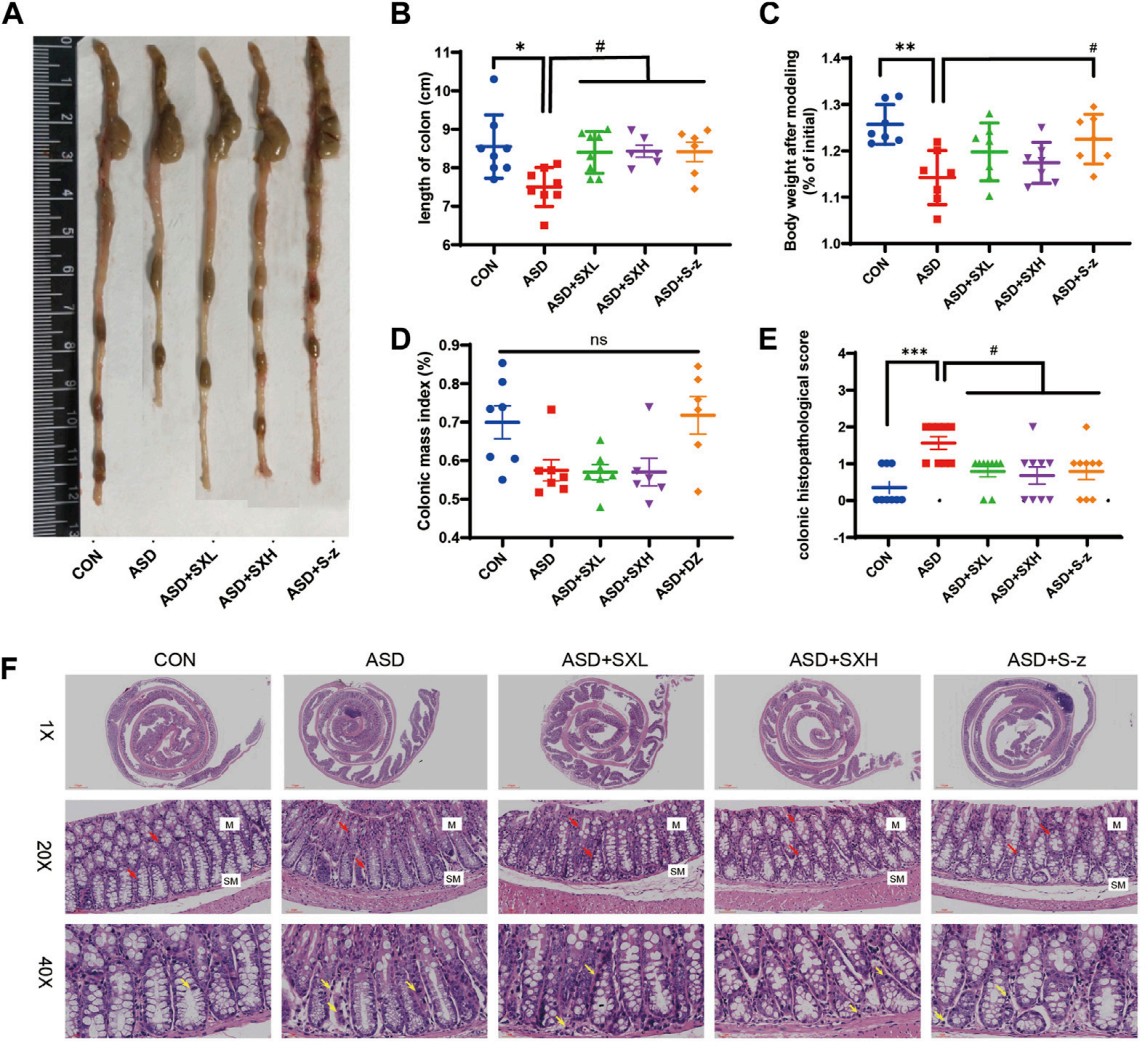
SX alleviates acute sleep deprivation-induced histopathological injury in the colon. **(A)** Representative images show the lengths of colons harvested from the CON, ASD, ASD + SXL, ASD + SXH, and ASD + S-z groups of mice. **(B)** Quantitative data shows the colon lengths of mice belonging to the CON, ASD, ASD + SXL, ASD + SXH, and ASD + S-z groups. **(C)** Body weights of the CON, ASD, ASD + SXL, ASD + SXH, and ASD + S-z group mice after modeling. **(D)** Colon mass indices (%) (colonic weight/body weight) of the CON, ASD, ASD + SXL, ASD + SXH, and ASD + S-z groups of mice. **(E)** The colon histopathological scores of the CON, ASD, ASD + SXL, ASD + SXH, and ASD + S-z group mice. *p* < 0.05, ASD vs. SXL, SXH and S-z. **(F)** Representative H&E-stained images show the morphological changes in the colon mucosa of mice belonging to the CON, ASD, ASD + SXL, ASD + SXH, and ASD + S-z groups (×1, ×20, and ×40 magnification). M: mucosa; SM: submucosa; red arrow: multiple mucinous glands; yellow arrow: inflammatory cells. Data were expressed as means ± SEM. *n* = 6–7 per group. Statistical significance was determined by one-way ANOVA; ^*^ *p* < 0.05 vs. control group; ^#^ *p* < 0.05 vs. ASD group; ns, no significant.

In the present study, we established an acute sleep deprivation model in C57BL/6J mice using the horizontal bar-style automated sleep deprivation system (Figure 1A). The ASD group mice gradually developed clinical symptoms of gastrointestinal dysfunction such as undigested stool, passivity, irritability, decreased appetite, disordered hair, and decreased body mass (*p <* 0.05, Figures 1D, E; Supplementary Tables S1–S9). The fasting blood glucose (FBG) levels in the ASD group mice were significantly higher than the CON group mice (*p <* 0.01, Figures 1F, G; Supplementary Tables S1–S9), but these effects were partially reversed in the ASD + SXH/SXL (high and low dose) and the ASD + S-z group mice (Figures 1F, G; Supplementary Tables S1–S9). The ASD group mice showed significant body weight loss compared to the CON group mice, but the body weights of the ASD + SXH/SXL (high and low dose) were not significantly higher than the ASD group mice (Figure 2C). Furthermore, fasting blood glucose (FBG) levels were significantly higher in the ASD group mice compared to the CON group, but were significantly lower in the ASD + SXH/SXL (high and low dose) and the ASD + S-z group mice compared to the ASD group (Figure 1G).

### 3.2 SX alleviates ASD-induced colon injury, inflammation, and dysfunction

The ASD group mice showed symptoms associated with gastrointestinal dysfunction, including undigested food in the stools, decreased appetite, and lower body mass. Therefore, we analyzed the colon lengths from distinct groups of mice. The ASD group mice showed reduced colon length (Figure 2A) and weight loss compared to the CON group mice (*p* < 0.05, Figure 2B; *p* < 0.01; Figure 2C; Supplementary Tables S1–S9). This demonstrated that ASD induced reduction in colon length. However, there was no significant difference in the colon mass index (ratio of colon weight to body weight) between the ASD and CON group mice. Histopathological examination of the colon showed mild structural changes in the colon mucosa of the ASD group mice including loss of goblet cells and crypt, and moderate infiltration of inflammatory cells in the mucosal (M) and submucosal (SM) regions of the colon (Figure 2F). Histopathological scores for the colon were significantly higher for the mice in the ASD group compared to the CON group mice (*p <* 0.001, Figure 2E). Furthermore, mice in the ASD + SXH/SXL (high and low dose) and the ASD + S-z groups showed lower numbers of inflammatory cells in the submucosal region of the colon (Figure 2F). The histopathological scores for the colon in the mice belonging to the ASD + SXH/SXL (high and low dose) and the ASD + S-z groups were significantly lower than those in the ASD group mice (Figure 2E). This demonstrated that SX and S-z alleviated ASD-induced colon injury, inflammation, and dysfunction.

### 3.3 SX alleviates ASD-induced oxidative stress

**FIGURE 3:**
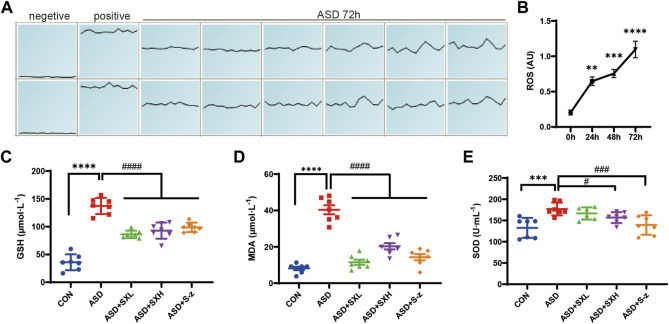
SX alleviates ASD-induced oxidative stress in the mouse serum. **(A) (A)**. ROS fluorescence intensity of blood cells after 72 h of ASD, waveforms are images obtained in “multi-point scanning” mode for each well, with the top and bottom wells being multiple wells. The average of each well was taken for statistical analysis. Positive control Rosup concentration was 100 μM. **(B)**. Fluorescence intensity before and after ASD was measured at 488 nm excitation wavelength and 535 nm emission wavelength, time point by time point (0, 24, 48, 72 h). **(B)** ROS levels in the blood cells of ASD group mice at 0, 24, 48, and 72 h. **(C)** Serum GSH levels in the CON, ASD, ASD + SXL, ASD + SXH, and ASD + S-z groups of mice. *p* < 0.0001, ASD vs. SXL, SXH, S-z. **(D)** Serum MDA levels in the CON, ASD, ASD + SXL, ASD + SXH, and ASD + S-z groups of mice. *p* < 0.0001, ASD vs. SXL, SXH, S-z. **(E)** Total SOD activity in the serum of CON, ASD, ASD + SXL, ASD + SXH, and ASD + S-z groups of mice. *p* < 0.05, ASD vs. SXH; *p* < 0.001 ASD vs. S-z. The data are represented as means ± SEM. *n* = 7 each group. Statistical significance was determined based on one-way ANOVA; ^****^ *p* < 0.0001, ^***^ *p* < 0.001, ^**^ *p* < 0.01 vs. CON group; ^####^ *p* < 0.0001, ^###^ *p* < 0.001, ^##^ *p* < 0.01, ^#^ *p* < 0.05 vs. ASD group.

Previous studies showed that colon length was reduced in mice with increased stress (Ono et al., 2022) and colitis (Ren et al., 2011; Wen et al., 2013; Zhang et al., 2018b). Thus, we investigated the correlation between oxidative stress and ASD modeling by estimating intracellular ROS levels in the blood cells using ROS-sensitive dye, DCFH-DA, with full wavelength fluorometric enzyme labeler, for three consecutive days after inducing SD. ROS levels showed gradual increase between days 1 and 3, and were significantly higher in the blood cells on day 3 (72 h) of modeling in the ASD group mice (*p* < 0.0001; Figure 3B). Here we presented the waveforms of the 72 h ROS fluorescence intensity (AU) assay (enzyme-labeled multi-point assay mode) (Figure 3A). Next, we analyzed the levels of ROS-related biomarkers, namely, GSH, SOD activity, and MDA, to determine the differences in oxidative stress product between the control and ASD groups, and if treatment with SX or S-z mitigated oxidative stress-induced colon damage in the ASD group mice. Compared with the CON group, the ASD group showed significantly higher levels of GSH (*p* < 0.0001; Figure 3C), MDA (*p* < 0.0001; Figure 3D), and SOD activity (*p* < 0.001; Figure 3E). This demonstrated that ASD induced oxidative stress. Furthermore, ASD + SXH/SXL and ASD + S-z mice showed significantly lower levels of GSH, MDA, and SOD activity compared with the ASD group mice (Figures 3C–E). These results demonstrated that SX and S-z protected against oxidative stress in the mice subjected to acute sleep deprivation.

### 3.4 Identification of SX target proteins based on molecular docking analysis

**FIGURE 4:**
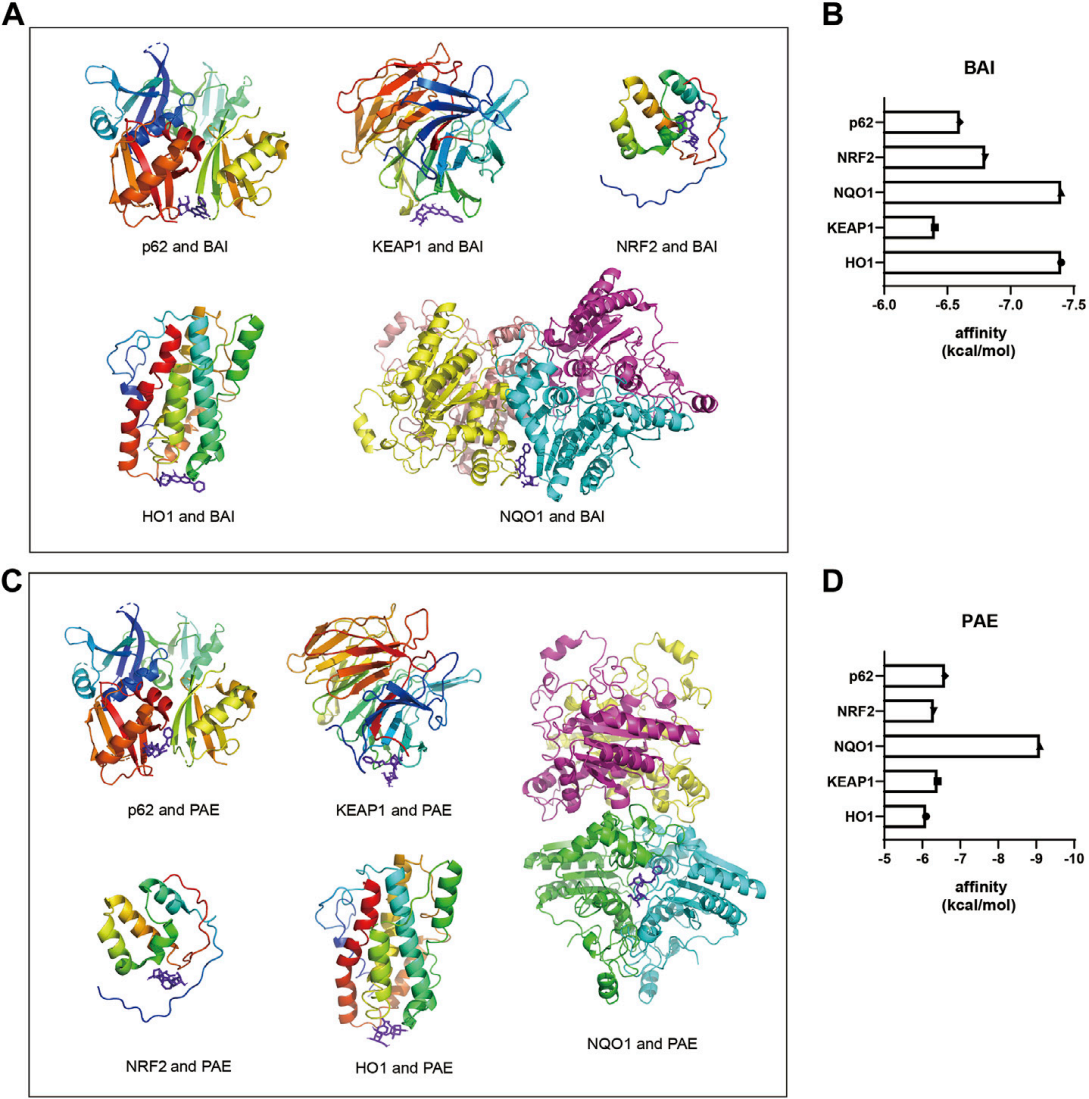
Molecular docking diagrams of BAI and PAE with p62, KEAP1, NRF2, HO1, and NQO1. **(A)** Molecular docking diagrams of BAI with p62, KEAP1, NRF2, HO1, and NQO1 proteins. **(B)** Binding affinity (kcal/mol) of BAI with p62, KEAP1, NRF2, HO1, and NQO1 proteins. **(C)** Molecular docking diagrams of PAE with p62, KEAP1, NRF2, HO1, and NQO1 proteins. **(D)** Binding affinity (kcal/mol) of PAE with p62, KEAP1, NRF2, HO1, and NQO1 proteins. BAI, Baicalin; PAE, Paeoniflorin.

To determine the potential target proteins of the SX Decoction, the sequences of the oxidative stress-related proteins such as p62, KEAP1, NRF2, NQO1, and HO1 were downloaded from the RCSB PDB archive. Their 3D structures were visualized using the PYMOL software and docked with the 3D structures of active ingredients of SX, baicalein (BAI) and paeoniflorin (PAE), using the AUTODOCK software to assess the binding affinities. Both BAI and PAE showed differential binding affinity with p62, KEAP1, NRF2, NQO1, and HO1 proteins (Figures 4A, C). NQO1 and HO1 showed strongest binding affinity with BAI (Figure 4B) and NQO1 showed strongest binding affinity with PAE (Figure 4D).

### 3.5 SX modulates ASD-induced NRF2 signaling pathway in the colon

**FIGURE 5:**
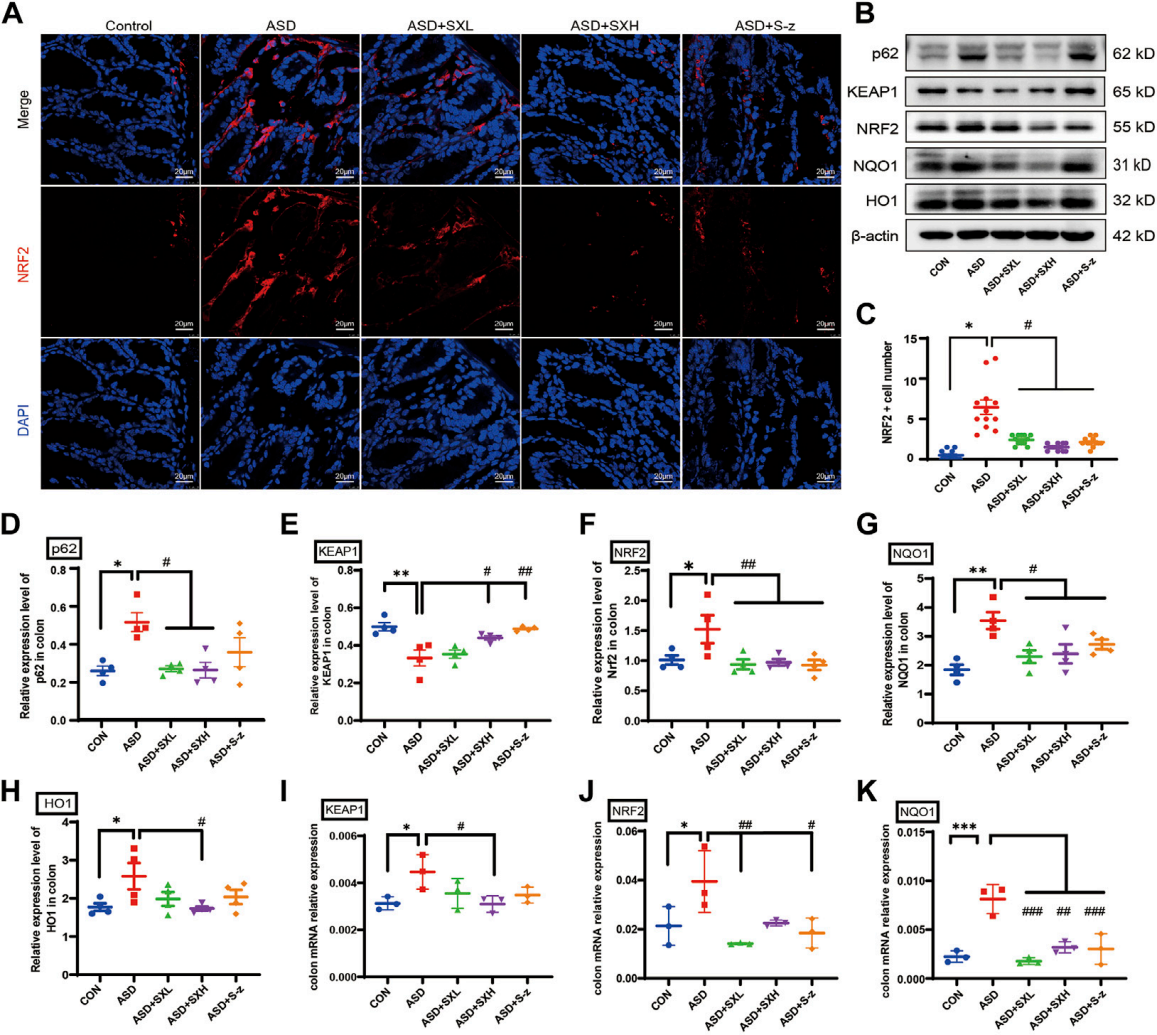
SX modulates ASD-induced oxidative stress in the colon through the p62/KEAP1/NRF2/HO1/NQO1 signaling pathway. **(A)** Representative immunofluorescence images show the localization of NRF2 protein in the colon sections based on staining with the fluorophore-labeled anti-NRF2 antibody (red fluorescence). The nuclei are stained with DAPI (blue fluorescence). **(B)** Representative western blot shows the expression levels of p62, KEAP1, NRF2, NQO1, HO1, and β-actin proteins in the colons of mice belonging to the CON, ASD, ASD + SXL, ASD + SXH, and ASD + S-z groups. β-actin was used as the internal loading control. The experiment was repeated three times. **(C)** The dot plot shows the numbers of NRF2 positive cells (based on immunofluorescence staining in A) in the colonic mucosa of mice belonging to the CON, ASD, ASD + SXL, ASD + SXH, and ASD + S-z groups. *p* < 0.05, ASD vs. SXL, SXH, and S-z. **(D–H)** The relative expression levels of p62, KEAP1, NRF2, NQO1, and HO1 proteins compared to the β-actin protein levels in the colons of mice belonging to the CON, ASD, ASD + SXL, ASD + SXH, and ASD + S-z groups. Note: for p62 in **(D)**, *p* < 0.05, ASD vs. SXL, SXH; *p* = 0.4302, ASD vs. S-z); for KEAP1 in **(E)**, *p* < 0.05 ASD vs. SXH; *p* < 0.01 vs. S-z); for NRF2 in **(F)**, *p* < 0.01, ASD vs. SXL, SXH, and S-z); for NQO1 in **(G)**, *p* < 0.05, ASD vs. SXL, SXH, and S-z; for HO1 in **(H)** *p* < 0.05, ASD vs. SXH); **(I–K)** qRT-PCR results show the transcript levels of KEAP1, NRF2, and NQO1 compared with the β-actin transcript levels. For KEAP1 in **(I)**, *p* < 0.05, ASD vs. SXH; for NRF2 in **(J)**, *p* < 0.01 ASD vs. SXL; *p* < 0.05, ASD vs. S-z; for NQO1 in **(K)**, *p* < 0.001 ASD vs. SXL and S-z; *p* < 0.01, ASD vs. SXH; KEAP1, Kelch-like ECH-associated protein 1; NRF2, nuclear factor erythroid 2-related factor 2; NQO1, NAD(P)H: quinone oxidoreductase 1; HO1, heme oxygenase 1; Data are expressed as means ± SEM. *n* = 4 per group. Statistical significance was determined by one-way ANOVA; ^*^ *p* < 0.05, ^**^ *p* < 0.01 vs. CON; ^#^ *p* < 0.05, ^##^ *p* < 0.01 vs. ASD. ns, no significant. Scale bar: 20 μm.

NRF2 is a key regulator of genes encoding antioxidant enzymes. During normal conditions, NRF2 is bound by KEAP1 in the cytoplasm and sequestered in an inactive state and targeted by the ubiquitin-proteasomal protein degradation pathway. KEAP1 is sensitive to redox changes and releases NRF2 when acted upon by ROS, which is generated by exogenous chemicals and oxidative damage. Subsequently, NRF2 translocated to the nucleus, binds to the ARE sequences of ROS-responsive genes, and activates transcription of the downstream oxidative stress response genes. Furthermore, NRF2 is also activated by accumulation of p62 (Jain et al., 2010; Tan et al., 2021). Therefore, nuclear translocation of NRF2 is a necessary step to activate the antioxidative mechanisms in the cells. Immunofluorescence staining of the colon sections demonstrated nuclear localization of NRF2 (red florescence) in the nucleus (cyan fluorescence of DAPI as the nucleus marker) of different groups (Figure 5A). The colon tissues from the ASD group showed increased nuclear localization of NRF2, but this effect was reversed by treatment with SX and S-z (Figure 5C). The expression levels of key oxidative stress-related proteins in the colon tissues were analyzed by qRT-PCR and western blotting. qRT-PCR results were confirmed by the western blotting results. qRT-PCR results showed significantly higher expression levels of KEAP1 (*p* < 0.05, Figure 5I), NRF2 (*p* < 0.05, Figure 5J), and NQO1 (*p* < 0.001, Figure 5K) transcripts in the ASD group compared to the CON group, but these effects were reversed in the ASD + SX mice (Figures 5I–K). Western blotting results also showed significantly higher expression levels of p62 (*p* < 0.05, Figure 5D), NRF2 (*p* < 0.05, Figure 5F), NQO1 (*p* < 0.01, Figure 5G), and HO1 (*p* < 0.05, Figure 5H) proteins and reduced expression levels of KEAP1 (*p* < 0.01, Figure 5E) in the ASD group compared to the CON group. Furthermore, SX and S-z treatment groups showed significant downregulation of p62, NRF2, NQO1, and HO1 proteins and upregulation of KEAP1 protein levels (Figures 5D–H). These results suggested that acute sleep deprivation enhanced the expression levels of NRF2-responsive antioxidative pathway genes in the mouse colon. Furthermore, our data demonstrated that SX was a promising therapy for the management of colon dysfunction and oxidative stress in subjects with acute sleep deprivation by targeting the p62/KEAP1/NRF2/HO1/NQO1 antioxidant pathway.

### 3.6 SX modulates ASD-induced antioxidative response in the colon through the NRF2 relative pathway

**FIGURE 6:**
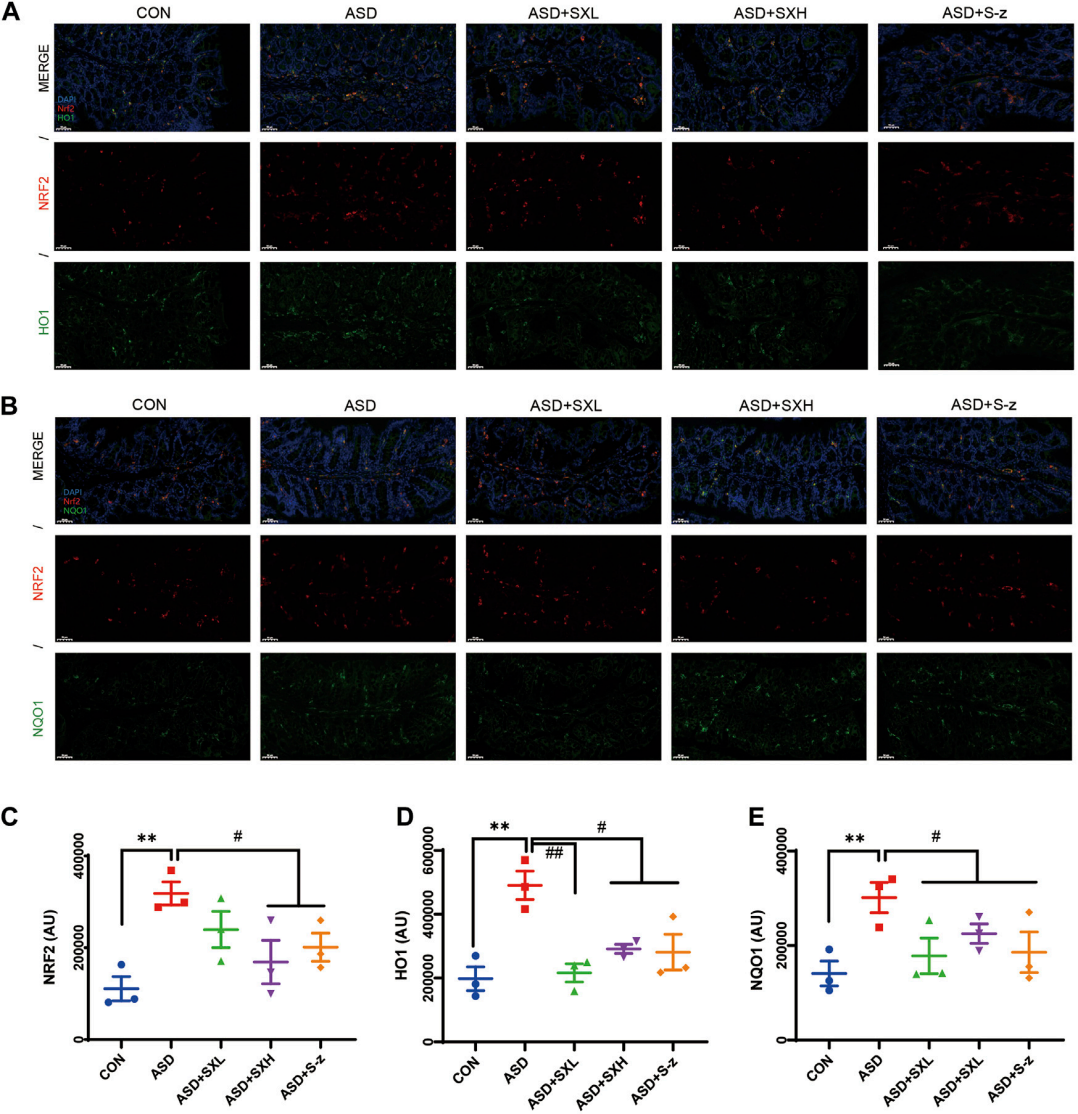
Immunofluorescence analysis of NRF2 (red), HO1 and NQO1 (green) in colon mucosal layers (×400 magnification). **(A)** Representative fluorescence confocal images show NRF2 and HO1 staining in the colon sections of mice belonging to the CON, ASD, ASD + SXL, ASD + SXH, and ASD + S-z groups. The nuclei were stained with DAPI (blue). **(B)** Representative fluorescence confocal images show NRF2 and NQO1 staining in the colon sections of mice belonging to the CON, ASD, ASD + SXL, ASD + SXH, and ASD + S-z groups. The nuclei were stained with DAPI (blue). **(C)** Quantitative analysis of NRF2 fluorescence. **(D)** Quantitative analysis of HO1 fluorescence. **(E)** Quantitative analysis of NQO1 fluorescence. The immunofluorescence signal intensity in the images was quantified using the ImageJ software. *n* = 3 per group. The experiment was repeated three times. The data are shown as mean ± S.E.M. ^**^ *p* < 0.01 vs. CON group; ^#^ *p* < 0.05, ^##^ *p* < 0.01 vs. ASD group. Scale bar: 50 μm.

Immunofluorescence microscopy was performed to confirm the above results. Colon sections from distinct groups of mice were subjected to immunofluorescence staining with antibodies against antioxidant pathway proteins. NRF2 (red fluorescence) and HO1 or NQO1 (green fluorescence) expression levels were higher in the colon sections from the ASD group mice compared to those from the CON group (Figures 6A, B), but these effects were reversed by SX or S-z treatment (Figures 6C–E). These data confirmed that SX modulated ASD-induced antioxidant response in the colon through the NRF2/NQO1/HO1 signaling pathway.

## 4 Discussion

Studies related to sleep disorders have generally focused on the central nervous system (CNS) because the suprachiasmatic nucleus of the hypothalamus represents the key center of sleep-wake regulation (Chen et al., 2018), and is regulated by the aminergic (Oikonomou et al., 2019; Faria et al., 2021) and the cholinergic brainstem and the hypothalamic system (Goldstein et al., 2018; Venner et al., 2019). However, sleep disturbance is a common comorbidity in subjects with inflammatory bowel diseases (IBD) such as Crohn’s disease (CD) and ulcerative colitis (UC) (Ananthakrishnan et al., 2014), and diabetes mellitus (Reutrakul and Van Cauter, 2018), and is linked to elevated risk of recurrence in subjects with CD (Ananthakrishnan et al., 2013). Sleep impairments are linked to alterations in the intestinal structure and functions, and are associated with symptoms such as altered stools and weight loss, possibly through disruption of the intestinal immune homeostasis and the intestinal microbiota (Gao et al., 2018). In this study, we treated the acute sleep-deprived mice with SX, which is used clinically for the treatment of sleep disorders, mood disorders, and gastrointestinal dysfunction, and investigated the potential mechanisms by which SX alleviated ASD-induced colon injury. Our study showed that ASD induced mild colon injury as evidenced by shortened colon length, altered colon morphology, moderate infiltration of inflammatory cells, and reduced numbers of cupped blood cells based on the H&E staining data. The shortening of colon length reduced digestive capacity because of decreased luminal surface area of the colon and the digestive glands and intestinal flora. Furthermore, our study showed that SX treatment significantly restored the length of the colon and the structure of the mucosal layer.

Oxidative phosphorylation in the mitochondria is a major site of ATP and ROS production in all cells. Physiological levels of ROS act as signaling molecules to maintain physiological functions. However, excessive production of ROS induces oxidative stress that extensively damages DNA (Srinivas et al., 2019; Tan et al., 2020), proteins (Gavazzi and Faury, 2021), and lipids (Farmer and Mueller, 2013). Sleep disturbances trigger a stress response and cause an imbalance between ROS production and antioxidant mechanisms, thereby resulting in persistently high intracellular ROS levels (Pandey and Kar, 2018). In our study, acute sleep-deprived mice demonstrate fasting hyperglycemia and increased levels of ROS, GSH, SOD, and MDA in the colon. This demonstrated that ASD-induced oxidative stress promoted colon damage and dysfunction.

Oxidative stress can be treated with antioxidant drugs or changes in extrinsic factors including nutrition, lifestyle, and radiation. Furthermore, the majority of the ROS-mediated signaling pathways and ROS detoxifying enzymes are regulated by the stress response transcription factor NRF2 and the nuclear factor κB (NF-kB) (DeNicola et al., 2011; Vernier et al., 2020). These represent promising therapeutic targets.

During oxidative stress, oxidation of multiple cysteine residues in KEAP1 inhibits its ubiquitin ligase activity and interaction with NRF2. This releases NRF2, which translocated into the nucleus and transcribes several target genes (Hochmuth et al., 2011). Furthermore, p62 binds and targets KEAP1 in the NRF2-KEPA1 complex for degradation, thereby releasing NRF2 for translocation into the nucleus (Kapuy et al., 2018; Yen et al., 2020). NRF2 modulates the expression of several downstream antioxidative genes such as NQO1, HO1, and GSH, which protect cells and tissues from xenobiotic and oxidative stress (Hochmuth et al., 2011). Our study confirmed previous reports that acute sleep deprivation promoted expression of antioxidant genes that protect against oxidative stress (Koutsoumparis et al., 2022). Our results showed that ASD induced transcription and translation of p62, KEAP1, NRF2, HO1, and NQO1 in the mouse colon. This demonstrated that ASD induced a significant antioxidant response in the mouse colon to manage excessive ROS levels. This caused significant changes in colon structure and functions. Therefore, our study showed that ASD activated the antioxidant response through the p62/KEAP1/NRF2/HO1/NQO1 signaling pathway. ASD also induced reduction in the levels of KEAP1 transcripts and protein.

SX contains promising drug candidates that may prevent and treat colitis and reduce inflammation-related gastrointestinal dysfunction, such as Salvia miltiorrhiza (Wen et al., 2013). In this study, we investigated the affinity of SX-major active components, baicalin (BAI) and paeoniflorin (PAE) (Zhang et al., 2022), and oxidative stress response proteins using molecular docking techniques. Our results demonstrated high affinity binding between SX-related components such as BAI and PAE and the antioxidant response proteins, NQO1, HO1, p62, NRF2, and KEAP1. Previous studies have shown that BAI could regulating the NRF2/KEAP1 through both KEAP1 non-dependent and -dependent pathways (Qin et al., 2014). These results were further confirmed by the RT-PCR and western blotting results. In this study, we used S-z as a positive drug control. S-z is a GABA-A receptor agonist that is currently used as a hypnotic drug and is associated with adverse effects such as dry mouth, dizziness, and drowsiness (Boyle et al., 2012; Yoshida et al., 2019; Carter et al., 2020). A high dose of S-z alone or in combination with its metabolites induces oxidative stress in the erythrocytes (Chan, 2014). In our study, the relative changes in the levels of p62, HO1, and NQO1 proteins were significantly higher with SX compared to S-z. This demonstrated that SX was a potent drug against oxidative stress in ASD. In a future study, we plan to validate the protective effects of SX against ASD-induced colon injury by using a mouse model of ASD combined with colon-specific knockout of NRF2. Furthermore, using CETSA or SPR will provide a deeper understanding on the direct interaction between main ingredients of SX and molecules, this will be addressed in future studies.

## 5 Conclusion

**FIGURE 7:**
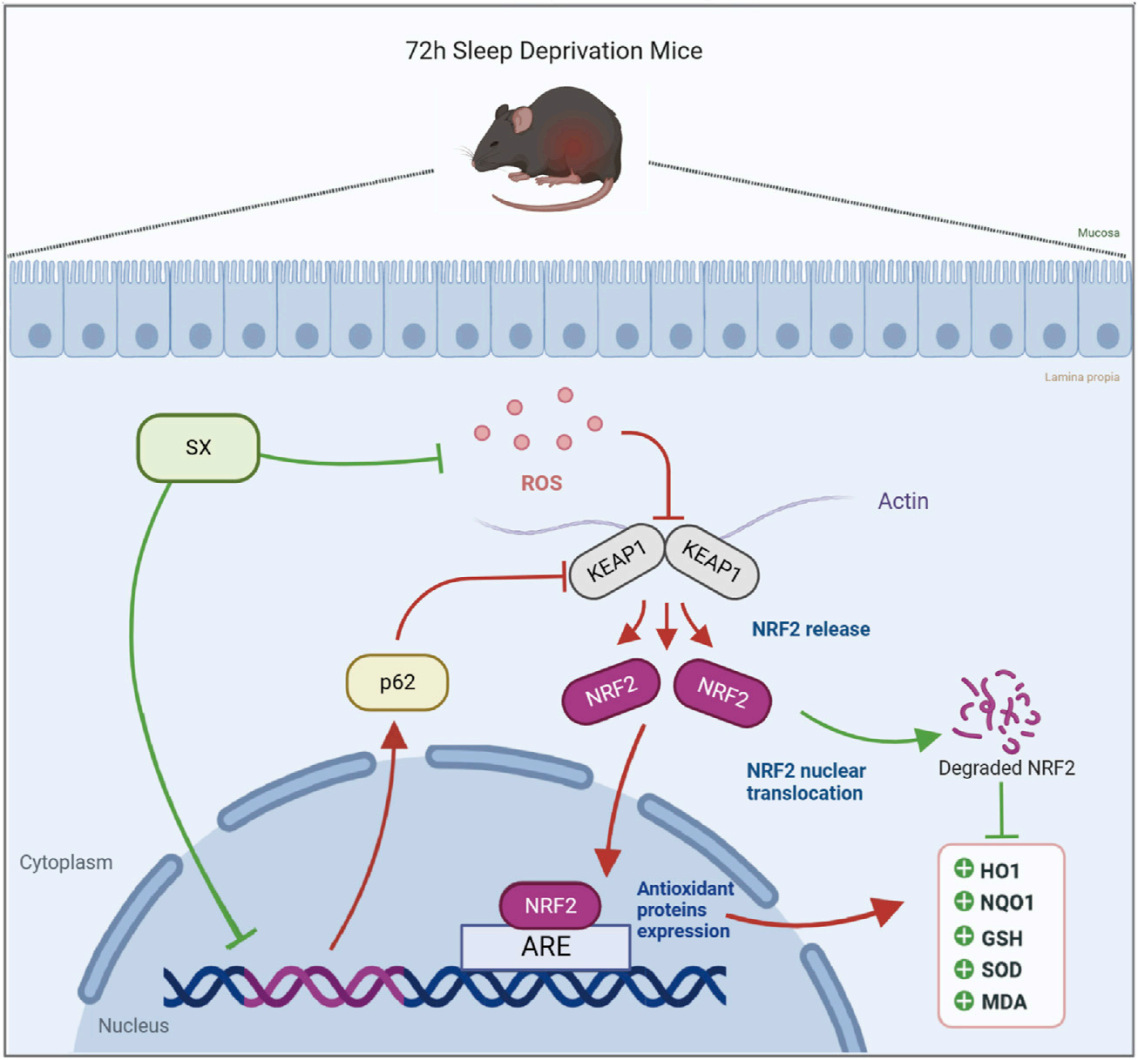
Diagrammatic representation shows the mechanism by which SX alleviates ASD-induced oxidative stress and colon injury.

This study demonstrated that acute sleep deprivation induced damage to the colon mucosal layer by increasing the ROS levels. Furthermore, we demonstrated that SX decoction significantly ameliorated ASD-induced colon injury by suppressing the p62/KEAP1/NRF2/HO1/NQO1 signaling pathway, which is activated in response to ASD-induced oxidative stress (Figure 7). Therefore, SX decoction is a promising therapeutic strategy for the treatment of SD-induced oxidative stress and colon injury.

## Data Availability

The original contributions presented in the study are included in the article/Supplementary Materials, further inquiries can be directed to the corresponding authors.
