# Corrigendum: Shu-Xie decoction alleviates oxidative stress and colon injury in acute sleep-deprived mice by suppressing p62/KEAP1/NRF2/HO1/NQO1 signaling

**DOI:** 10.3389/fphar.2023.1199204

**Published:** 2023-05-30

**Authors:** Mengyuan Wang, Bo Li, Yijiang Liu, Mengting Zhang, Caoxin Huang, Teng Cai, Yibing Jia, Xiaoqing Huang, Hongfei Ke, Suhuan Liu, Shuyu Yang

**Affiliations:** ^1^ Research Studio of Traditional Chinese Medicine, The First Affiliated Hospital of Xiamen University, School of Medicine, Xiamen University, Xiamen, Fujian, China; ^2^ The First Affiliated Hospital of Xiamen University, School of Medicine, Xiamen University, Xiamen, Fujian, China

**Keywords:** sleep deprivation, oxidative stress, NRF2, traditional Chinese medicine, ROS

In the published article, there was an error in Figure 6 as published. The pictures for ASD + S-z groups in Figure 6 were erroneously presented after being redrawn and submitted. The corrected Figure 6 and its caption appear below.

**FIGURE 6:**
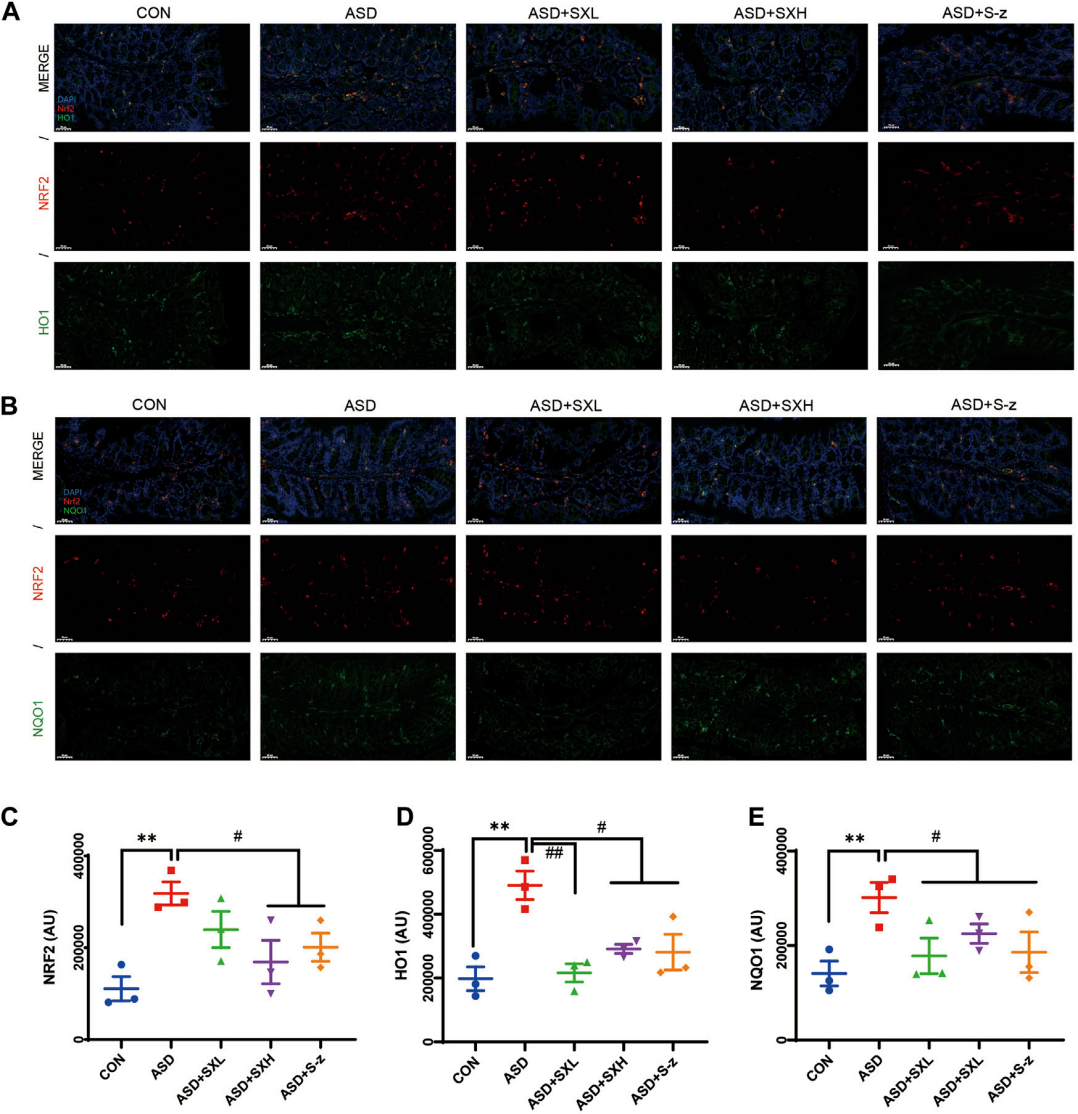
Immunofluorescence analysis of NRF2 (red), HO1 and NQO1 (green) in colon mucosal layers (×400 magnification). **(A)** Representative fluorescence confocal images show NRF2 and HO1 staining in the colon sections of mice belonging to the CON, ASD, ASD + SXL, ASD + SXH, and ASD + S-z groups. The nuclei were stained with DAPI (blue). **(B)** Representative fluorescence confocal images show NRF2 and NQO1 staining in the colon sections of mice belonging to the CON, ASD, ASD + SXL, ASD + SXH, and ASD + S-z groups. The nuclei were stained with DAPI (blue). **(C)** Quantitative analysis of NRF2 fluorescence. **(D)** Quantitative analysis of HO1 fluorescence. **(E)** Quantitative analysis of NQO1 fluorescence. The immunofluorescence signal intensity in the images was quantified using the ImageJ software. n = 3 per group. The experiment was repeated three times. The data are shown as mean ± S.E.M. ^**^ *p* < 0.01 vs CON group; ^#^ *p* < 0.05, ^##^ *p* < 0.01 vs ASD group. Scale bar: 50 μm.

The authors apologize for this error and state that this does not change the scientific conclusions of the article in any way. The original article has been updated.

