# Supplementary material for: Shu-Xie decoction alleviates oxidative stress and colon injury in acute sleep-deprived mice by suppressing p62/KEAP1/NRF2/HO1/NQO1 signaling
Source: Front Pharmacol. 2023 Feb 6;14:1107507. doi: 10.3389/fphar.2023.1107507 (PMC9939528; doi:10.3389/fphar.2023.1107507)
Supplement: Supplementary file 1 [file DataSheet1.PDF]

Shu-Xie Decoction Alleviated Acute Sleep Deprivation-Induced Colon Oxidant stress via p62 / KEAP1 / Nrf2 / HO1 / NQO1 Signaling

Mengyuan Wang[1#], Bo Li[1#], Yijiang Liu[2], Mengting Zhang[1], Caoxin Huang[3], Hongfei Ke[1], Suanhuan Liu[4*], Shuyu Yang[1*]

[1] Research Studio of Traditional Chinese Medicine, The First Affiliated Hospital of Xiamen University, School of Medicine, Xiamen University, Xiamen 361003, Fujian, China

[2] The First Affiliated Hospital of Xiamen University, School of Medicine, Xiamen University, Xiamen 361003, Fujian, China

[3] Xiamen Diabetes Institute, The First Affiliated Hospital of Xiamen University, School of Medicine, Xiamen University, Xiamen 361003, Fujian, China

[4] Research Center for Translational Medicine, The First Affiliated Hospital of Xiamen University, School of Medicine, Xiamen University, Xiamen 361003, Fujian, China

* Correspondence:

Shuyu Yang and Suhuan Liu; xmyangshuyu@126.com; liusuhuan@xmu.edu.cn

[#] Mengyuan Wang and Bo Li contributed equally to this manuscript.

Supplementary Material

1 Supplementary Tables

Table S1 Composition of ShuXie Decoction (SX, one dose).

Herb	Family	Part used	Concocting method	Amount used (g)
Semen Ziziphi Spinosae (Ziziphus jujube Mill.var.spinosa (Bunge) Hu ex H.F.Chou)	Rhamnaceae	Seed	Frying	15
Poria (Poria cocos (Schw.) Wolf)	Polyporaceae	Sclerotium	Cutting	15
Rhizoma Chuanxiong (Ligusticum anthriscoides 'Chuanxiong')	Umbelliferae	Root	Cutting	10
Scutellariae Radix (Scutellaria baicalensis Georgi)	Lamiaceae	Root	Cutting	15
Pinelliae Rhizoma (Pinellia ternate (Thunb.) Breit.)	Araceae	Tuber	Cooked (ginger, alum)	10
Anemarrhenae Rhizoma (Anemarrhena asphodeloides Bge.)	Liliaceae	Root	Cutting	10
Aurantii Fructus Immaturus (Citrus aurantiuml L.)	Rutaceae	Fruit	Cutting	10
Schisandrae Chinesis Fructus (Schisandra chinensis (Turcz.) Baill.)	Magnoliaceae	Fruit	Smash	10
Bambusae Caulis in Taenias (Bambusa tuldoides Munro)	Gramineae	Mid. layer of stem	Cutting	10
Paeoniae Radix Alba (Paeonia tacti lora Pall.)	Ranunculaceae	Root	Frying	15

Table S2 Antibodies employed for Western blotting analyses.

	Company	CAS	Dilution
KEAP1 (D1G10) Rabbit mAb	Cell Signaling Technology	7705	1:1000
Nrf2 (A-10) Mouse mAb	Santa Cruz Biotechnology	sc-365949	1:500
Heme Oxygenase 1 (F-4) Mouse mAb	Santa Cruz Biotechnology	sc-390991	1:500
NQO1 (A180) Mouse mAb	Santa Cruz Biotechnology	sc-32793	1:500
Anti-p62 (SQSTM1) pAb	MBL	PM045	1:1000
Beta-Actin Mouse mAb	SIGMA	A3854	1:5000
Goat Anti-Mouse IgG (H+L), HRP	ThermoFisher	31430	1:10000
Goat Anti-Rabbit IgG (H+L), HRP	ThermoFisher	31460	1:10000

Table S3 Primer sequences for SYBR Green-based RT-PCR analyses.

Primer		Sequences (5' to 3')
β-actin	Forward	GTACCACCATGTACCCAGGC
β-actin	Reverse	AACGCAGCTCAGTAACAGTCC
KEAP1	Forward	TCGAAGGCATCCACCCTAAG
KEAP1	Reverse	CTCGAACCACGCTGTCAATCT
Nrf2	Forward	GCCGTCTCCTCTCGGATATG
Nrf2	Reverse	CTGGCGAACAGGGGATGTG
NQO1	Forward	TCCAGACTCCGATCATCAAGC
NQO1	Reverse	GCTCATGGTGTTCAGAATTGTGT

Table S4 The consumption volume of drinking water in mice of each group. (mL)

**Day**	**CON**	**ASD**	**ASD+SXL**	**ASD+SXH**	**ASD+S-z**
**7**	50.0	45.0	45.0	45.0	45.0
**14**	51.0	40.0	45.0	50.0	50.0
**15**	52.0	50.0	50.0	52.0	51.0
**16**	54.0	50.0	50.0	52.0	48.0
**17**	54.0	38.0	38.0	38.0	40.0
**Mean**	52.20	44.60	45.60	47.40	46.80
**SD**	1.789	5.550	4.930	5.983	4.438

Table S5 The consumption of feed in mice of each group. (g)

**Day**	**CON**	**ASD**	**ASD+SXL**	**ASD+SXH**	**ASD+S-z**
**7**	45.9	47	48.3	49.2	43.4
**14**	47	35.6	46.6	34.2	40.9
**15**	46.5	44.5	41.9	40.4	46.5
**16**	47.8	43.1	48.2	46.8	43.7
**17**	45.1	36.9	42.3	41.9	40.1
**Mean**	46.46	41.42	45.46	42.50	42.92
**SD**	1.031	4.943	3.144	5.853	2.534

Table S6 Body weight after modeling (% of initial) in mice of each group. (%)

Sample / Group	1	2	3	4	5	6	7	Mean ± SD
CON	1.314	1.237	1.260	1.215	1.230	1.223	1.317	1.257 ± 0.04273
ASD	1.097	1.116	1.153	1.157	1.219	1.200	1.052	1.142 ± 0.05839*
ASD+SXL	1.102	1.221	1.227	1.166	1.280	1.143	1.242	1.197 ± 0.06230#
ASD+SXH	1.179	1.121	1.250	1.200	1.132	1.153	1.184	1.174 ± 0.04411
ASD+S-z	1.226	1.189	1.189	1.262	1.295	1.144	1.268	1.225 ± 0.05356##

*$\rho < 0.05$ compared to CON group; #$\rho < 0.05$, ##$\rho < 0.01$ compared to ASD group.

Table S7 The fasting blood glucose levels after modeling in mice of each group. (mmol/L)

Sample / Group	1	2	3	4	5	6	7	Mean ± SD
CON	7.2	6.7	6.9	7.1	6.2	6.1	6.2	6.629 ± 0.4608
ASD	8.8	9.2	9.4	9.9	7.8	8.2	8.8	8.871 ± 0.7135*
ASD+SXL	6.7	7.8	8.0	7.3	8.5	7.2	8.3	7.686 ± 0.6466#
ASD+SXH	7.2	7.2	6.9	8.5	7.9	7.8	7.5	7.571 ± 0.5407#
ASD+S-z	7.6	7.0	7.0	7.4	6.9	7.2	6.8	7.129 ± 0.2870#

Note: Values are presented as Mean ± Standard Deviation. The ANOVA for repeated measures showed significant fasting blood glucose levels effects between different group.

*$\rho < 0.05$ compared to CON group; #$\rho < 0.05$ compared to ASD group.

Table S8 The colon length of each group after modeling.

Sample / Group	1	2	3	4	5	6	7	8	Mean ± SD
CON	8.3	9.1	8.5	8.5	10.3	8.0	8.0	7.7	8.550 ± 0.8246
ASD	7.3	8.1	7.6	7.8	7.4	8.0	7.3	6.5	7.500 ± 0.5071
ASD+SXL	7.7	8.8	7.7	9.0	8.3	8.9	8.8	8.0	8.400 ± 0.5451
ASD+SXH	8.8	9.0	8.2	8.4	8.4	-	-	-	8.560 ± 0.3286
ASD+S-z	7.5	7.9	9.0	8.9	8.6	-	-	-	8.380 ± 0.6535

Note: '-' means loss of the values unwillingly. Values are presented as Mean ± Standard Deviation. The ANOVA for repeated measures showed no significant colon index effects between different group.

Table S9 The colon length and colon index of each group after modeling. (%)

Sample / Group	1	2	3	4	5	6	7	Mean ± SD
CON	0.605	0.804	0.735	0.385	0.610	0.854	0.550	0.6489 ± 0.1614
ASD	0.557	0.575	0.732	0.517	0.580	0.561	0.653	0.5965 ± 0.0726
ASD+SXL	0.602	0.486	0.554	0.487	0.530	0.739	0.732	0.5899 ± 0.1072
ASD+SXH	0.845	0.519	0.506	0.514	0.623	0.694	0.598	0.6143 ± 0.1230
ASD+S-z	0.713	0.553	0.667	0.584	0.496	0.475	0.639	0.5895 ± 0.0882

Note: Colon length and colon index, defined as colon weight in a 5.0-cm-long colon cylinder divided by total body weight. Values are presented as Mean ± Standard Deviation. The ANOVA for repeated measures showed no

significant colon index effects between different group.

2 Supplementary Pictures

Picture S1 HE staining of colonic sections of each group.

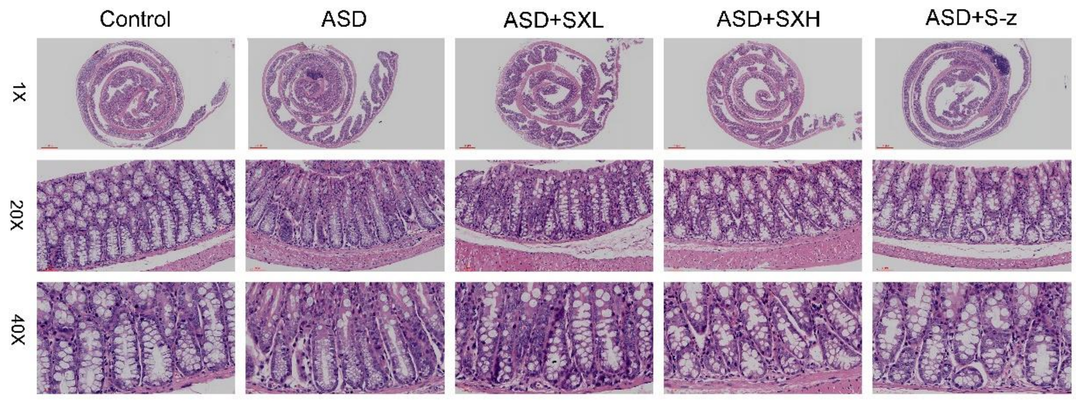

Picture S2 Weight changes of each group.

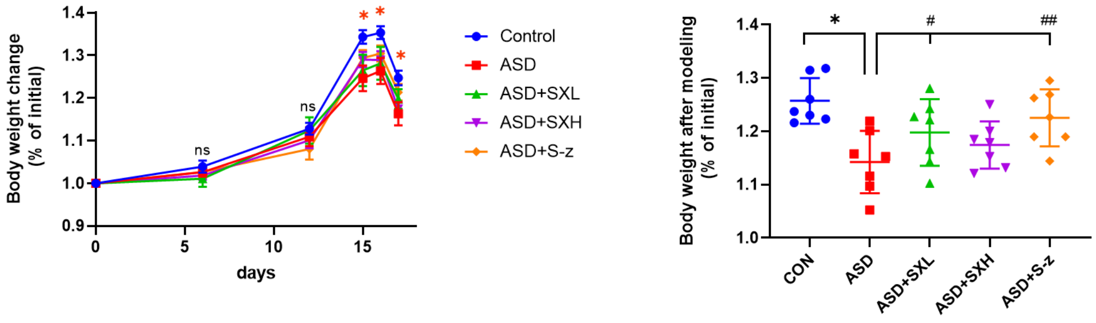

Picture S3 Fasting Glucose of each group.

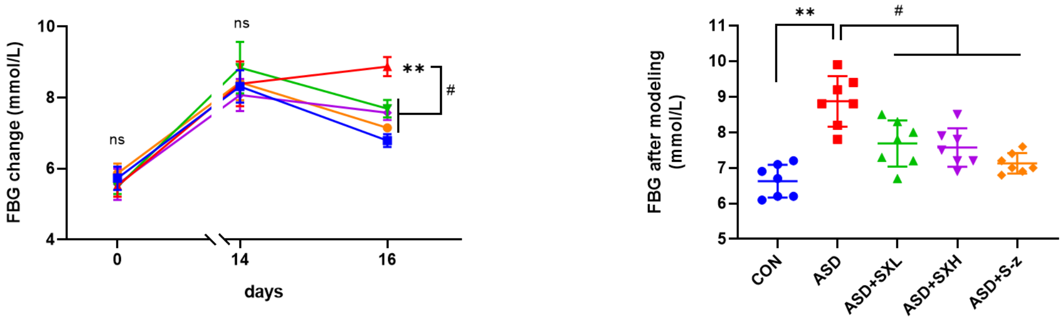

Picture S4 Western blot membranes of each group.

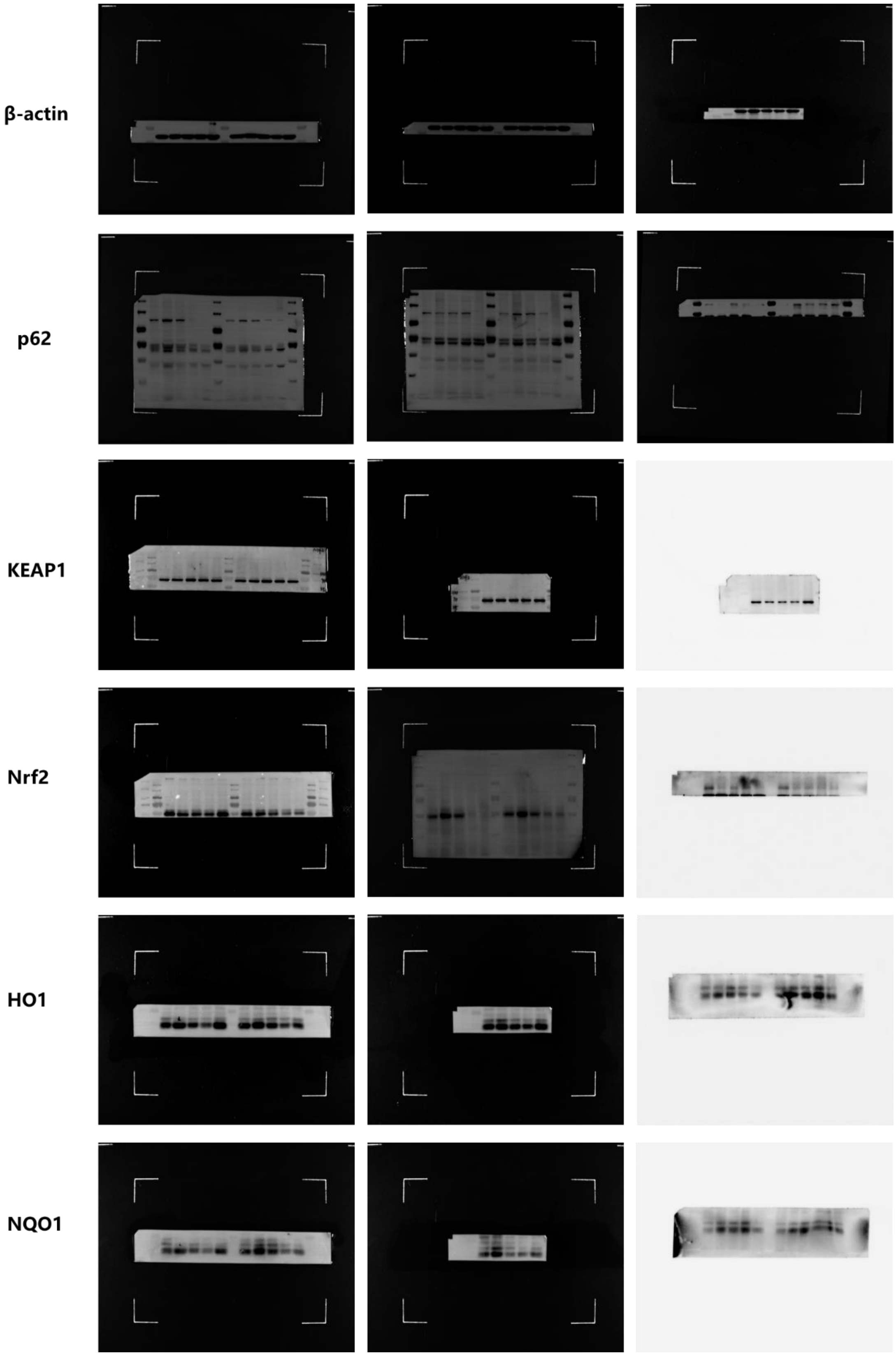
β-actin
p62
KEAP1
Nrf2
HO1
NQO1
